# Physics-Informed Data-Driven Prediction of 2D Normal Strain Field in Concrete Structures

**DOI:** 10.3390/s22197190

**Published:** 2022-09-22

**Authors:** Mauricio Pereira, Branko Glisic

**Affiliations:** Department of Civil and Environmental Engineering, Princeton University, Princeton, NJ 08544, USA

**Keywords:** predictive modeling, creep and shrinkage, structural health monitoring, long-term structural behavior, physics-informed machine learning, optical fibers, fiber bragg grating

## Abstract

Concrete exhibits time-dependent long-term behavior driven by creep and shrinkage. These rheological effects are difficult to predict due to their stochastic nature and dependence on loading history. Existing empirical models used to predict rheological effects are fitted to databases composed largely of laboratory tests of limited time span and that do not capture differential rheological effects. A numerical model is typically required for application of empirical constitutive models to real structures. Notwithstanding this, the optimal parameters for the laboratory databases are not necessarily ideal for a specific structure. Data-driven approaches using structural health monitoring data have shown promise towards accurate prediction of long-term time-dependent behavior in concrete structures, but current approaches require different model parameters for each sensor and do not leverage geometry and loading. In this work, a physics-informed data-driven approach for long-term prediction of 2D normal strain field in prestressed concrete structures is introduced. The method employs a simplified analytical model of the structure, a data-driven model for prediction of the temperature field, and embedding of neural networks into rheological time-functions. In contrast to previous approaches, the model is trained on multiple sensors at once and enables the estimation of the strain evolution at any point of interest in the longitudinal section of the structure, capturing differential rheological effects.

## 1. Introduction

Concrete is the most used man-made material, and concrete infrastructures, such as bridges, buildings, and dams, form the backbone of modern societies [1]. Concrete infrastructure is expected to be serviceable for several decades, and its life may be extended for social, economic, or even symbolic reasons.

However, concrete structures present long-term, time-dependent behavior due to creep and shrinkage. Creep is the delayed strain evolution observed under sustained material stress. Shrinkage is the change in volume observed in concrete associated with water losses and capillary forces [1,2,3]. Accurate prediction of these rheological effects is important for distinguishing expected structural behavior from long-term damage processes, such as corrosion [4], differential settlement [5,6], or scouring [7], the latter a leading cause of collapse of structures in North America [8], as well as for improved assessment of prestress losses and long-term performance of civil structures [9,10].

Multiple empirical constitutive models for prediction of creep and shrinkage in concrete exist in the literature [2,11,12,13,14]. However, the majority of these empirical models are set to minimize the average fitting error to databases of creep and shrinkage laboratory tests, largely composed of experiments performed on prismatic specimen lasting less than five years, far below the expected life of most civil structures. Long-term creep and shrinkage information are even more limited for novel concrete compositions, richer in admixtures and with lower water-to-cement ratio in which autogenous shrinkage can be significant [2].

Furthermore, the creep and shrinkage laboratory experiments present limitations. For example, because specimens are center-loaded and axisymmetric, experiments do not capture differential creep and shrinkage. However, rheological effects depend on temperature, humidity, and loading that are not uniform throughout the real structure, inducing differential creep and shrinkage. Furthermore, in reinforced concrete elements with nonuniform rebar distribution, a curvature is induced by their restraining effect on shrinkage [2,15,16]. Thus, prediction of cross-sectional averages is not sufficient to fully capture the long-term time-dependent behavior of concrete structures.

Current application of empirical constitutive creep and shrinkage models towards long-term prediction of structural behavior requires a numerical model, commonly a finite element model (FEM), able to account for the concrete–rebar interaction, and judicious application of the creep compliance to appropriate segments of the structure [2,17]. Numerical modeling is further complicated by the need of specifying appropriate aging and steel relaxation models [18,19,20], which impacts the long-term time-dependent behavior of concrete structures. Since creep and shrinkage depend on temperature and humidity, to consider differential effects associated with temperature and humidity gradients requires solving for the temperature and humidity fields in the structure, which can be difficult if appropriate boundary and initial conditions are uncertain. State-of-the-art comprehensive numerical modeling includes thermo-chemical effects that impact creep and shrinkage [21,22,23], but such numerical approaches are computationally intensive even for small domains, and still require the use of empirical compliance, shrinkage, and chemical reaction models. The level of geometric detail that includes aggregate geometry, used in the state-of-the-art numerical models is not typically available for real structures. Instead, smoothed strain and stress fields (e.g., obtained by considering average concrete properties in FEM) are sufficient to capture broader structural behavior.

Furthermore, because the existing empirical creep and shrinkage models were developed largely for structural design (e.g., [13]) and not structural health monitoring (SHM), the compliance and shrinkage models with preset parameters are unlikely to be optimal to predict the long-term behavior of a specific monitored structure.

For example, it is possible to improve the prediction accuracy of creep and shrinkage laboratory experiments by updating model parameters with information from short-term (e.g., few weeks) experiments [2,24], and Bayesian methods have been proposed to incorporate short-term data towards improved long-term prediction of deflection and creep [17,25].

Data-driven approaches have also been explored towards improved prediction of long-term time-dependent behavior in concrete. Proposed methods include the use of artificial neural networks (NNs) for prediction of shrinkage [26], and of thermal and long-term effects in concrete structures [27], with encouraging results. However, these approaches are applied to laboratory or synthetic data only. In general, scaling to real world data are not straightforward, since real data present noise, anomalies (e.g., jumps and drift), and irregularly missing intervals [28]. These characteristics prevent the application of standard machine learning approaches that require complete data [29,30].

SHM systems can provide in-situ data from the structure, with potential for improved long-term prediction accuracy. For example, long gauge fiber optics sensors (FOS) based on fiber Bragg grating (FBG) can measure temperature and average strain at the sensor location, along its axis. In contrast to laboratory experiments, in-situ strain measurements capture a multitude of effects present in the structure, including seasonal temperature variations and the resulting long-term time-dependent behavior due to creep, shrinkage, concrete–rebar interaction, aging, and steel relaxation. For example, recently, Ref. [31] applied convolutional neural networks (CNNs) towards prediction of long-term behavior with excellent results. Even though physics-informed machine learning show great potential [32], the current data-driven models present in the literature do not leverage the structure’s geometry and loading and, in the case of a structure with multiple sensors, require a different model for each sensor, potentially missing broader structural behavior that emerge if sensors are considered holistically, such as differential rheological behavior.

The aim of this work is to estimate the total long-term time-dependent 2D normal strain field in prestressed concrete beam structures. Our work contributes to the literature in prediction of long-term time-dependent behavior by introducing a physics-informed data-driven method that holistically incorporates multiple sensors in the structure and predicts differential rheological effects over multiple years. Our method employs multiple in-situ long-gauge discrete point strain and temperature measurements to train a physics-informed data-driven model and predict strain and temperature measurements at any other point in the longitudinal section, effectively providing (smooth) estimates of the 2D normal strain and temperature fields. In virtue of this, with the same model, reasonably good prediction accuracy is obtained even at unobserved (during training) points. The physics aspect of the model is introduced via simplified analytical modeling of strain in the structure, while the data-driven aspect is introduced vian NNs within the model to predict the temperature field and incorporate spatiotemporal variations of rheological effects. Hence, our work introduces an original integration between strain analysis and machine learning towards prediction of long-term structural behavior and, to the best of the author’s knowledge, is the first work to address long-term strain field prediction. The method is applied to a pedestrian bridge, the Streicker Bridge.

The paper is organized as follows: In Section 2, the Streicker Bridge and sensor characteristics are introduced; and the development of the simplified model, NN architectures, and training policy are presented; In Section 3, strain predictions and other outputs of the model are presented and discussed; in Section 4, the concluding remarks and future work are given.

## 2. Materials and Methods

In this section, the structure under study is introduced, the development of the predictive model from the simplified analytical model to the embedding of NNs is presented, together with the proposed model training, validation, and testing strategy.

### 2.1. Streicker Bridge

**Figure 1:**
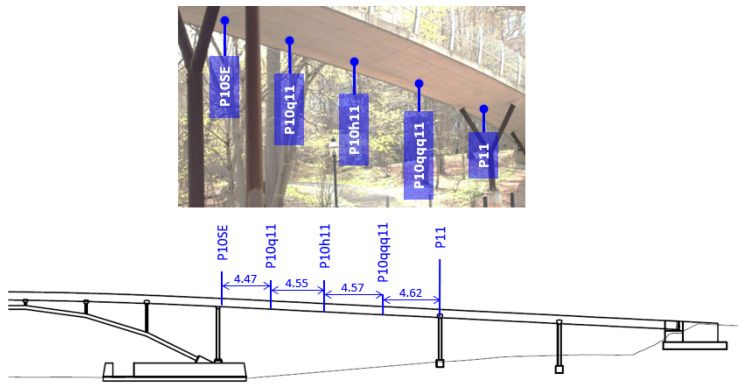
Segment P10-P11 of Streicker Bridge (all distances in meters). Inset shows a picture of the bridge span of interest with instrumented sections highlighted.

The Streicker Bridge is a pedestrian bridge located in Princeton, New Jersey. Its main span consists of a deck-stiffened arch, with varying cross-section, while the approaching legs are curved continuous girders of constant cross-section, supported by Y-shaped steel columns. The bridge is instrumented with fiber optics strain and temperature sensors based on FBG in the main span and the southeast leg [33]. This study focuses on a segment of the southeast leg, shown in Figure 1 [34], but the overall methodology can be applied to other segments of the bridge. Typical characteristics of the sensors are given in Table 1. Sensors are installed at multiple positions across the span, denominated P10SE, P10q11, P10h11, P10qqq11, and P11, as shown in Figure 1, with their axis parallel to the centroid line, such that strain measurements correspond to the normal strain component in the direction of the centroid line (i.e., perpendicular to the cross-section). At each instrumented position along the span, two sensors are present in the vertical plane containing the vertical principal axis of the cross-section, one at the upper location (up sensor) and another at the bottom location (down sensor). For example, the up sensor at position P10SE is referred to as P10SE-U, while the bottom sensor as P10SE-D. Strain and temperature data collected over seven years were used for the training, validation, and testing of the proposed model. For a comprehensive description of the monitoring system, and the bridge, the interested reader is referred to [33].

### 2.2. Total Strain Change Model

At a damage-free position $x=\left[x,y\right]\in \Omega \subset R^{2}$, where *x* is the position along the span, *y* is the position along the beam depth with respect to the centroid of stiffness, and Ω represents the longitudinal section between P10 and P11 (see Figure 1); the total normal strain measured at time *t*, in days, is

(1) $$\varepsilon_{tot}^{x,t,t^{\prime },t^{\prime \prime }}=\varepsilon_{T}^{x,t}+\varepsilon_{e}^{x,t}+\varepsilon_{R}^{x,t,t^{\prime },t^{\prime \prime }},$$

where $\varepsilon_{T}^{x,t}$, $\varepsilon_{e}^{x,t}$, $\varepsilon_{R}^{x,t,t^{\prime },t^{\prime \prime }}$ are the thermal, elastic, and rheological strain, respectively, at position x for times $t>t^{\prime \prime }\geq t^{\prime }$, where $t^{\prime }$ is the time of prestressing and $t^{\prime \prime }$ is the time of form and cover removal. The superscript corresponds to the arguments of the function.

The elastic strain is

(2) $$\varepsilon_{e}^{x,t}=\varepsilon_{e,T}^{x,t}+\varepsilon_{e,P}^{x,t}+\varepsilon_{e,W}^{x},$$

where $\varepsilon_{e,T}^{x,t}$ is the elastic response to thermal expansion and contraction due to mechanical constraints in indeterminate structures, $\varepsilon_{e,P}^{x,t}$ is the elastic strain due to prestressing, and $\varepsilon_{e,W}^{x}$ is the elastic strain due to self-weight.

The rheological strain can be written as

(3) $$\varepsilon_{R}^{x,t,t^{\prime },t^{\prime \prime }}=\varepsilon_{Cr,P}^{x,t,t^{\prime }}+\varepsilon_{Cr,W}^{x,t,t^{\prime \prime }}+\varepsilon_{Sh}^{x,t,t^{\prime \prime }},$$

where $\varepsilon_{Cr,P}^{x,t,t^{\prime }}$, $\varepsilon_{Cr,W}^{x,t,t^{\prime \prime }}$ are the creep strain due to prestressing and self-weight, respectively, and $\varepsilon_{Sh}^{x,t,t^{\prime \prime }}$ is the shrinkage strain. We consider that long-term creep due to thermally generated stresses can be neglected as thermal stresses average out due to seasonal periodicity of temperature changes. The prestressing and self-weight creep strains are, respectively,

(4) $$\varepsilon_{Cr,P}^{x,t,t^{\prime }}=\varepsilon_{e,P}^{x,t^{\prime }}\varphi^{x,t,t^{\prime }}+\int_{t^{\prime }}^{t}\varphi^{x,t,u}d\varepsilon_{e,P}^{x,u},$$

(5) $$\varepsilon_{Cr,W}^{x,t,t^{\prime \prime }}=\varepsilon_{e,W}^{x,t^{\prime \prime }}\varphi^{x,t,t^{\prime \prime }},$$

where $\varphi^{x,t,u}$ is the creep coefficient at position x, time *t* for loading at time *u*, and the integral term in Equation (4) corresponds to creep recovery associated with prestress losses and aging of concrete.

The shrinkage strain is

(6) $$\varepsilon_{Sh}^{x,t,t^{\prime \prime }}={\bar{\varepsilon }}_{Sh}^{x}\psi^{x,t,t^{\prime \prime }},$$

where ${\bar{\varepsilon }}_{Sh}^{x}$ is the notional shrinkage strain, and $\psi^{x,t,t^{\prime \prime }}$ is the shrinkage coefficient for onset of drying at time $t^{\prime \prime }$.

Let $\xi^{t}$ be a generic strain component. Then, let us define the operator

(7) $$\delta \xi^{t}=\xi^{t}-\xi^{t^{\prime \prime }}=\int_{t^{\prime \prime }}^{t}d\xi^{u},$$

considering $\xi^{t}$ is differentiable.

Introducing Equations (2) and (3) into (1), and applying the operator (7) gives the change in total strain after form removal,

(8) $$\delta \varepsilon_{tot}^{x,t,t^{\prime },t^{\prime \prime }}=\delta \varepsilon_{e,T}^{x,t}+\delta \varepsilon_{e,P}^{x,t}+\delta \varepsilon_{e,W}^{x}+\delta \varepsilon_{Cr,P}^{x,t,t^{\prime }}+\delta \varepsilon_{Cr,W}^{x,t,t^{\prime \prime }}+\delta \varepsilon_{Sh}^{x,t,t^{\prime \prime }}.$$

Applying the definitions given in Equations (7), the strain changes associated with prestressing are

(9) $$\delta \varepsilon_{e,P}^{x,t}+\delta \varepsilon_{Cr,P}^{x,t,t^{\prime }}=\varepsilon_{e,P}^{x,t^{\prime }}\left(\varphi^{t,t^{\prime }}-\varphi^{x,t^{\prime \prime },t^{\prime }}\right)+{\bar{\varepsilon }}_{P_{\theta }}^{x}\theta^{x,t,t^{\prime \prime }},$$

with

(10) $${\bar{\varepsilon }}_{P_{\theta }}^{x}\theta^{x,t,t^{\prime \prime }}=\int_{t^{\prime \prime }}^{t}\left(1+\varphi^{x,t,u}\right)d\varepsilon_{e,P}^{x,u},$$

where the notional prestress strain ${\bar{\varepsilon }}_{P_{\theta }}^{x}$ and the coefficient $\theta^{x,t,t^{\prime \prime }}$ represent a tentative approximation of the evolution of the prestress strain changes plus the creep recovery due to prestress loss and aging given by the integral on the right-hand side.

Let also

(11) $$\delta \varepsilon_{T}^{x,t}=\alpha \Delta T^{x,t},$$

(12) $$\delta \varepsilon_{e,T}^{x,t}=-\kappa_{e}\Delta T^{x,t},$$

where α is the coefficient of thermal expansion (CTE), and $\kappa_{e}$ is a constant related to the degree of mechanical constraint. That is, the thermal strain and elastic response to thermal strain depend on

(13) $$\Delta T^{x,t}=T^{x,t}-T^{x,t^{\prime \prime }},$$

the local change in temperature. Then, let

(14) $$\delta \varepsilon_{TD}^{x,t}=\eta \Delta T^{x,t},$$

be the change in the temperature-dependent strain component, with

(15) $$\eta =\alpha -\kappa_{e},$$

the *apparent* CTE.

Then, using Equations (5), (6), (9), and (14), the change in total strain is

(16) $$\begin{array}{cc} \delta \varepsilon_{tot}^{x,t,t^{\prime },t^{\prime \prime }} & =\eta \Delta T^{x,t}+\varepsilon_{e,P}^{x,t^{\prime }}\left(\varphi^{x,t,t^{\prime }}-\varphi^{x,t^{\prime \prime },t^{\prime }}\right)\\ & +\varepsilon_{e,W}^{x,t^{\prime \prime }}\varphi^{x,t,t^{\prime \prime }}+\varepsilon_{P_{\theta }}^{x}\theta^{x,t,t^{\prime \prime }}+{\bar{\varepsilon }}_{Sh}^{x}\psi^{x,t,t^{\prime \prime }}. \end{array}$$

Consider that the time-dependent coefficients can be approximated by scaling of the creep coefficient that is

(17) $$\theta^{x,t,t^{\prime \prime }}=\rho_{\theta }\varphi^{x,t,t^{\prime \prime }},$$

(18) $$\psi^{x,t,t^{\prime \prime }}=\rho_{\psi }\varphi^{x,t,t^{\prime \prime }},$$

where $\rho_{\theta }$ and $\rho_{\psi }$ are positive scaling coefficients. Using Equations (17) and (18) in Equation (16),

(19) $$\begin{array}{cc} \delta \varepsilon_{tot}^{x,t,t^{\prime },t^{\prime \prime }} & =\eta \Delta T^{x,t}+\varepsilon_{e,P}^{x,t^{\prime }}\left(\varphi^{x,t,t^{\prime }}-\varphi^{x,t^{\prime \prime },t^{\prime }}\right)\\ & +\left(\varepsilon_{e,W}^{x,t^{\prime \prime }}+\rho_{\theta }\varepsilon_{P_{\theta }}^{x}+\rho_{\psi }{\bar{\varepsilon }}_{Sh}^{x}\right)\varphi^{x,t,t^{\prime \prime }}. \end{array}$$

Equation (19) could be further simplified if the time of form removal $t^{\prime \prime }$ was approximately equal to the prestressing time $t^{\prime }$. However, for Streicker Bridge, the difference is significant, circa 10 days. Still, we can impose the condition $t^{\prime }=t^{\prime \prime }$, and reinterpret the creep coefficient $\varphi^{x,t,t^{\prime }}$ as a notional rheological coefficient that yields an equivalent long-term strain evolution. Notice that aging is also implicitly accounted for by the notional rheological coefficient. Since under this consideration we set $t^{\prime }=t^{\prime \prime }$ (i.e., take the form removal date as the reference), and since $t^{\prime \prime }$ is fixed, the day of prestressing $t^{\prime }$ and of form removal $t^{\prime \prime }$ are omitted as function arguments henceforth. Then, Equation (19) becomes

(20) $$\delta \varepsilon_{tot}^{x,t}=\eta \Delta T^{x,t}+{\bar{\varepsilon }}^{x}\varphi^{x,t},$$

with

(21) $${\bar{\varepsilon }}^{x}=\varepsilon_{e,P}^{x}+\varepsilon_{e,W}^{x}+\rho_{\theta }\varepsilon_{P_{\theta }}^{x}+\rho_{\psi }{\bar{\varepsilon }}_{Sh}^{x},$$

the apparent static strain.

The prestressing force and its subsequent drop affect both the shortening and bending of the beam, while self-weight causes bending of the beam. The shrinkage strain is typically considered as an average term that evolves uniformly over the cross-section [11]. However, in the presence of asymmetrically distributed rebars or non-uniform temperature and humidity distribution over the cross-section depth, a curvature is induced by differential shrinkage. Here, the shrinkage-induced curvature is modeled as being the result of an apparent bending moment, and the longitudinal shortening due to shrinkage as the result of an apparent normal force. Hence, the apparent static strain is modeled as

(22) $${\bar{\varepsilon }}^{x}=\frac{\bar{N}}{EA}+\frac{{\bar{M}}^{x}y}{EI},$$

where $\bar{N}$, ${\bar{M}}^{x}$ are, respectively, the apparent normal force and apparent bending moment. Furthermore, consider that the bending moment ${\bar{M}}^{x}$ is caused by a constant distributed load across the span. Then, the static strain is of the form

(23) $${\bar{\varepsilon }}^{x}\left[w_{\varepsilon }\right]=\left[x^{2}y,xy,y,1\right]\cdot w_{\varepsilon }$$

with $w_{\varepsilon }$ parameters to be learned from the data.

Let the rheological strain coefficient be parameterized by $w_{R}$, that is,

(24) $$\varphi^{x,t}=\varphi^{x,t}\left[w_{R}\right].$$

Substituting Equations (23) and (24) into Equation (20),

(25) $$\delta \varepsilon_{tot}^{x,t}\left[w_{\varepsilon ,R}\right]=\eta \Delta T^{x,t}+{\bar{\varepsilon }}^{x}\left[w_{\varepsilon }\right]\cdot \varphi^{x,t}\left[w_{R}\right],$$

where $w_{\varepsilon ,R}=\left[w_{\varepsilon },w_{R}\right]$.

**Figure 2:**
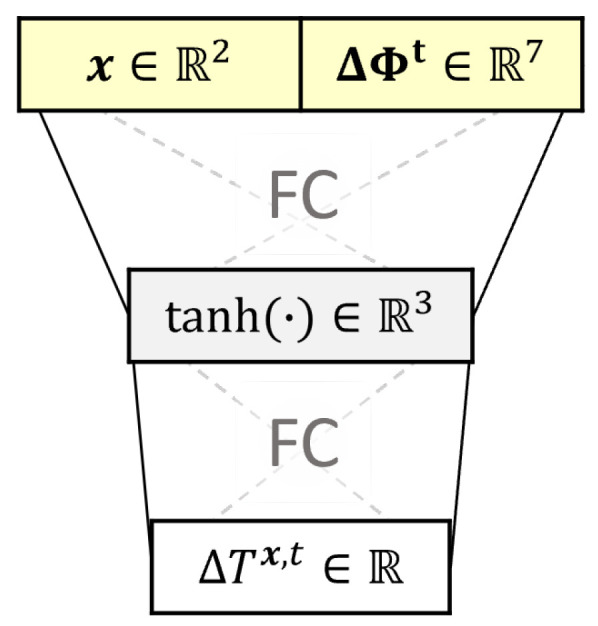
Architecture of $\Delta T_{NN}^{x,\Delta \Phi^{t}}$.

In principle, Equation (25) can be evaluated at any point x∈Ω, thus providing the normal strain field in Ω. However, the change in temperature $\Delta T^{x,t}$ is measured only at a finite set of points $P={\left\{x_{i}\right\}}_{i=1}^{N_{m}}$, which prevents the evaluation of Equation (25) everywhere in Ω. To address this limitation, the change in temperature is modeled as

(26) $$\Delta T^{x,t}=\Delta T_{NN}^{x,\Delta \Phi^{t}}\left[w_{\Delta T}\right],$$

where $\Delta T_{NN}^{x,\Delta \Phi^{t}}$ is an NN with parameters $w_{\Delta T}$ that take as input the position x and change in local environment conditions

(27) $$\Delta \Phi^{t}=\left[\Delta T_{air,max}^{t-2:t},\Delta T_{air,min}^{t-2:t},\Delta Q^{t}\right],$$

with $\Delta Q^{t}$ the change in solar radiation, and

(28) $$\Delta T_{air,max}^{t-2:t}=\left[\Delta T_{air,max}^{t},\Delta T_{air,max}^{t-1},\Delta T_{air,max}^{t-2}\right],$$

(29) $$\Delta T_{air,min}^{t-2:t}=\left[\Delta T_{air,min}^{t},\Delta T_{air,min}^{t-1},\Delta T_{air,min}^{t-2}\right],$$

the 3-day history of the local maximum and minimum change in air temperature, respectively. The NN architecture used is shown in Figure 2. It takes as input the position $x\in R^{2}$ in the cross-section, and the change in local environment conditions $\Delta \Phi^{t}\in R^{7}$, followed by a fully connected (FC) layer with the hyperbolic tangent as activation function that outputs a vector in $R^{3}$, followed by another FC layer that outputs the change in temperature prediction $\Delta T^{x,t}$. The architecture is kept simple due to the limited amount of training data, since there is one data point per day and only one year of data are used to train the model, and is inspired by previous work by the authors [35].

**Figure 3:**
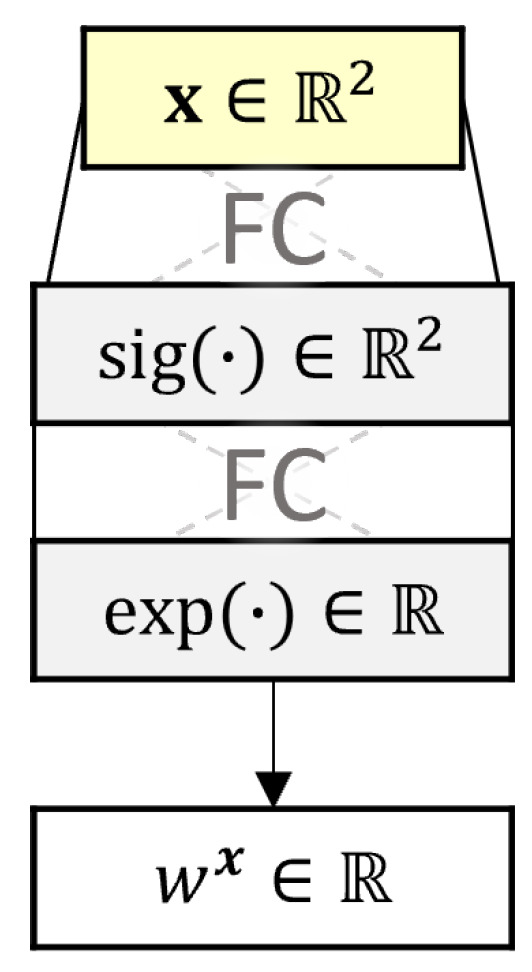
Architecture of $w_{NN}^{x}$.

The notional rheological strain evolution function was initially taken as

(30) $$\begin{array}{cc} \varphi^{x,t}\left[w_{R}\right] & =w_{R,1}\log\left(1+\Delta t^{2}\right)+\frac{w_{R,2}}{1+{\left(\frac{w_{R,3}}{t}\right)}^{w_{R,4}}}\\ \; & -w_{R,1}\log\left(1+{\left(\Delta t^{\prime \prime }\right)}^{2}\right)-\frac{w_{R,2}}{1+{\left(\frac{w_{R,3}}{t^{\prime \prime }}\right)}^{w_{R,4}}} \end{array},$$

where $w_{R,i}$ is the i-th component of $w_{R}$, and $\Delta t=t-t^{\prime \prime }$. Notice that it is independent of the position x. The model above is obtained by modifying and removing some components of the B4 compliance model [14] and considering a change with respect to time $t^{\prime \prime }$. However, introducing a dependency on the position x increased prediction accuracy. This is achieved by substituting the constant $w_{R,1}$ by an NN $w_{NN}^{x}\left[w_{\varphi }\right]$, with weights and biases $w_{\varphi }$ that take as input the position x. The architecture of the NN is shown in Figure 3. The NN takes as input the position $x\in R^{2}$ in the cross-section, followed by an FC layer with a sigmoid activation function that outputs a vector in $R^{2}$. However, the coefficient should be allowed to take any positive value, so the following FC layers use an exponential activation function to enforce positive values. The output corresponds to a real positive value. Similarly to the NN used for temperature prediction, the architecture is kept small due to a limited amount of data.

Thus, the notional rheological strain evolution function is

(31) $$\begin{array}{cc} \varphi_{NN}^{x,t}\left[w_{R}\right] & =w_{NN}^{x}\left[w_{\varphi }\right]\log\left(\frac{1+\Delta t^{2}}{1+{\left(\Delta t^{\prime \prime }\right)}^{2}}\right)\\ \; & +\frac{w_{R,2}}{1+{\left(\frac{w_{R,3}}{t}\right)}^{w_{R,4}}}-\frac{w_{R,2}}{1+{\left(\frac{w_{R,3}}{t^{\prime \prime }}\right)}^{w_{R,4}}} \end{array},$$

with $w_{R}=\left[w_{\varphi },w_{R,2},w_{R,3},w_{R,4}\right]$, and the subscript on the left-hand side is added to emphasize that an NN is embedded in the coefficient.

Substituting Equation (26) and (31) into Equation (25),

(32) $$\begin{array}{cc} \delta \varepsilon_{tot}^{x,t,\Delta \Phi^{t}}\left[w_{\varepsilon ,R},w_{\Delta T}\right] & =\eta \Delta T_{NN}^{x,\Delta \Phi^{t}}\left[w_{\Delta T}\right]\\ \; & +{\bar{\varepsilon }}^{x}\left[w_{\varepsilon }\right]\cdot \varphi_{NN}^{x,t}\left[w_{R}\right]. \end{array}$$

Equation (32) provides a decomposition of the total strain change in the temperature-dependent component and the rheological, time-dependent component,

(33) $$\begin{array}{c} \delta \varepsilon_{R}^{x,t}\left[w_{\varepsilon ,R}\right]={\bar{\varepsilon }}^{x}\left[w_{\varepsilon }\right]\cdot \varphi_{NN}^{x,t}\left[w_{R}\right]. \end{array}$$

Furthermore, ${\bar{\varepsilon }}^{x}$ and $\varphi_{NN}^{x,t}$ can also be retrieved separately. However, each is only known up to a scaling factor, since neither the true scaling of the rheological coefficient nor that of the apparent static strain are known. For example, the same rheological strain $\delta \varepsilon_{R}^{x,t}\left[w_{\varepsilon ,R}\right]$ would be found by doubling the rheological coefficient $\varphi_{NN}^{x,t}$ and halving the apparent static strain ${\bar{\varepsilon }}^{x}$. Nonetheless, these functions can still reveal where the apparent static strain is higher or smaller with respect to the average, and the relative spatiotemporal variation of the notional rheological coefficient. Therefore, let

(34) $${\tilde{\xi }}^{x}=\frac{{\bar{\varepsilon }}^{x}}{\max_{x}\left|{\bar{\varepsilon }}^{x}\right|},$$

(35) $${\tilde{\varphi }}_{NN}^{x,t}=\frac{\varphi_{NN}^{x,t}}{\max_{x,t}\left|\varphi_{NN}^{x,t}\right|},$$

be the normalized apparent static strain and the normalized rheological coefficient, respectively.

Substituting Equation (33) into Equation (32),

(36) $$\delta \varepsilon_{tot}^{x,t,\Delta \Phi^{t}}\left[w_{\varepsilon ,R},w_{\Delta T}\right]=\eta \Delta T_{NN}^{x,\Delta \Phi^{t}}\left[w_{\Delta T}\right]+\delta \varepsilon_{R}^{x,t}\left[w_{\varepsilon ,R}\right].$$

Equation (36) is the model of the change in total strain after form removal, with parameters $w_{\varepsilon ,R},w_{\Delta T}$ learned from measured data that can be evaluated everywhere in Ω, hence yielding the evolution of the normal strain field.

### 2.3. Model Training

The goal is to obtain a model capable of predicting the strain field at any point in Ω. However, real structures are typically instrumented at a finite number of positions *N*. Thus, data from only $N_{obs}<N$ observed positions are considered to train the model, and the remaining instrumented positions are considered to test the model. Notice that validation data (i.e., data used to prevent overfitting to the training data) must come from the observed positions only, in accordance with the real life target scenario in which no data are available from other positions in the structure.

**Figure 4:**
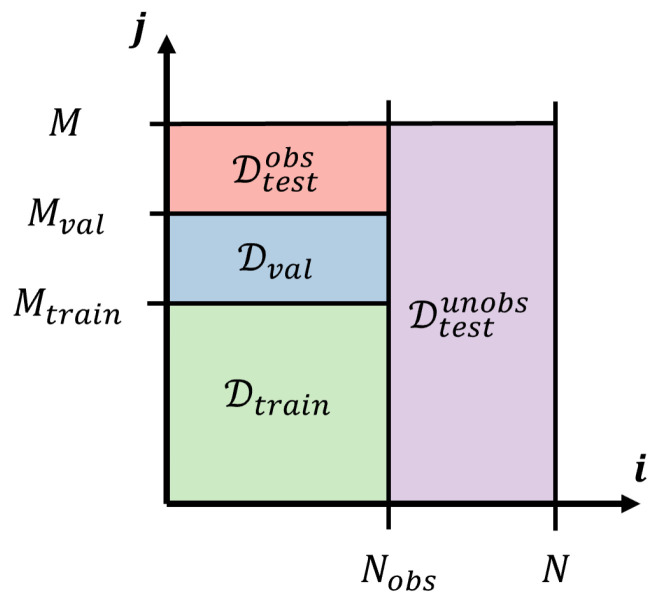
Diagram showing the training, validation, and test datasets.

Let

(37) $$D_{train}={\left\{\left(\delta \varepsilon_{m}^{x_{i},t_{j}},\Delta T_{m}^{x_{i},t_{j}},\Delta \Phi^{t_{j}}\right)\right\}}_{i=1,j=1}^{N_{obs},M_{train}},$$

(38) $$D_{val}={\left\{\left(\delta \varepsilon_{m}^{x_{i},t_{j}},\Delta T_{m}^{x_{i},t_{j}},\Delta \Phi^{t_{j}}\right)\right\}}_{i=1,j=M_{train}+1}^{N_{obs},M_{val}},$$

be the set of training and validation data, respectively, and

(39) $$D_{test}^{obs}={\left\{\left(\delta \varepsilon_{m}^{x_{i},t_{j}},\Delta T_{m}^{x_{i},t_{j}},\Delta \Phi^{t_{j}}\right)\right\}}_{i=1,j=M_{val}+1}^{N_{obs},M},$$

(40) $$D_{test}^{unobs}={\left\{\left(\delta \varepsilon_{m}^{x_{i},t_{j}},\Delta T_{m}^{x_{i},t_{j}},\Delta \Phi^{t_{j}}\right)\right\}}_{i=N_{obs}+1,j=1}^{N,M},$$

be the set of test data at *observed* and *unobserved* positions, respectively, and

(41) $$D_{test}=D_{test}^{obs}\cup D_{test}^{unobs},$$

be the set of *all* test data, with $\delta \varepsilon_{m}^{x_{i},t_{j}}$, $\Delta T_{m}^{x_{i},t_{j}}$, $\Delta \Phi^{t_{j}}$ the measured change in total strain, temperature, and local environmental conditions, respectively, at position $x_{i}$ at time $t_{j}$, $M_{train}$, and $M_{val}$, the indices corresponding to the end of training and validation, respectively, and *M* the total number of points in time, with $M_{train}<M_{val}<M$. The datasets defined in Equations (37)–(40) are illustrated in Figure 4. The training data are used to determine the model parameters, according to the optimization problems to be defined below, while the validation data are used to prevent overfitting via early stopping [36]. Notice that the test data do not inform the model in any capacity, meaning that the model is not trained or validated using data from unobserved positions. Therefore, predictions at unobserved positions correspond to full reconstruction of the expected change in strain.

The parameters $w_{\Delta T}$ of the temperature change model given by Equation (26) can be learned prior to the remaining parameters $w_{\varepsilon ,R}$ of the total strain change model, since temperature data from the structure are available as a target for the temperature change model. Then, the optimal weights $w_{\Delta T}^{*}$ are

(42) $$w_{\Delta T}^{*}=\underset{w_{\Delta T}}{\mathrm{argmin}}\sum_{i=1}^{N_{obs}}\sum_{j=1}^{M_{train}}{\left(\Delta T_{m}^{x_{i},t_{j}}-\Delta T_{NN}^{x_{i},\Delta \Phi^{t_{j}}}\left[w_{\Delta T}\right]\right)}^{2}.$$

Notice that the summations cover the data available in $D_{train}$. The minimization problem in Equation (42) is solved using the backpropagation algorithm with stochastic gradient descent (SGD) [37]. Furthermore, to prevent overfitting, the minimization is carried until the validation loss

(43) $$L_{\Delta T}^{val}=\sum_{i=1}^{N_{obs}}\sum_{j=M_{train}+1}^{M_{val}}{\left(\Delta T_{m}^{x_{i},t_{j}}-\Delta T_{NN}^{x_{i},\Delta \Phi^{t_{j}}}\left[w_{\Delta T}^{*}\right]\right)}^{2},$$

covering the data available in $D_{val}$, is at a minimum.

The optimal weights $w_{\varepsilon ,R}^{*}$ are

(44) $$w_{\varepsilon ,R}^{*}=\underset{w_{\varepsilon ,R}}{\mathrm{argmin}}\sum_{i=1}^{N_{obs}}\sum_{j=1}^{M_{train}}{\left(\delta \varepsilon_{m}^{x_{i},t_{j}}-\delta \varepsilon_{tot}^{x_{i},t_{j}}\left[w_{\varepsilon ,R},w_{\Delta T}^{*}\right]\right)}^{2}.$$

Similarly to the previous case, the minimization is performed using backpropagation with SGD until the validation loss

(45) $$L_{\varepsilon_{tot}}^{val}=\sum_{i=1}^{N_{obs}}\sum_{j=M_{train}+1}^{M_{val}}{\left(\delta \varepsilon_{m}^{x_{i},t_{j}}-\delta \varepsilon_{tot}^{x_{i},t_{j}}\left[w_{\varepsilon ,R}^{*},w_{\Delta T}^{*}\right]\right)}^{2},$$

is at a minimum.

Then, the predicted change in total strain at any desired position $x_{p}\in \Omega$ and time $t_{p}>t^{\prime \prime }$ is $\delta \varepsilon_{tot}^{x_{p},t_{p}}\left[w_{\varepsilon ,R}^{*},w_{\Delta T}^{*}\right]$.

## 3. Results

Acquired temperature and strain data covers a period of seven years. Both the temperature and strain models are trained on data covering the first year after construction, validated on data from the subsequent semester, and tested on the remaining 5.5 years. These datasets present several irregularly missing intervals, sometimes with months worth of data missing. It may be possible to train and validate such models using data collected over a shorter period of time if less data are missing. Test data at observed positions span several years after the train and validation data, while test data at unobserved positions include the entire time span. We show that good accuracy can be achieved at multiple positions, and that the predictions of the temperature and strain model enable the detection of anomalies in the data at some positions in the bridge.

**Figure 5:**
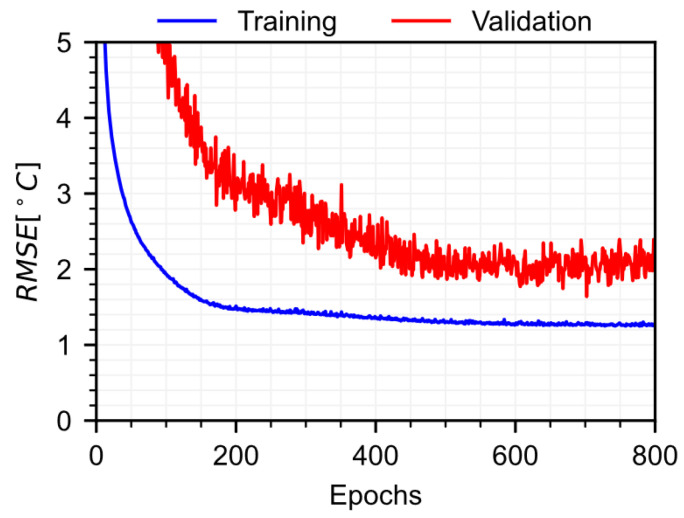
Training and validation losses for the temperature model.

The temperature model is trained for 5000 epochs with a learning rate of 0.001, using the Adam optimizer [37]. The training and validation losses of the temperature model are shown in Figure 5. The temperature model validation errors are around 2.5 ${}^{{}^{\circ}}$C, which is less than 15% of the typical temperature amplitude observed at Streicker Bridge. The strain model validation errors are around 50 με, about 10% of the strain magnitude typically observed at Streicker Bridge, which was sufficient for long-term anomaly detection. Part of the strain prediction errors are due to temperature prediction errors. Considering a CTE of 10 με${/}^{{}^{\circ}}$C, typical for concrete, suggests that around 50% of the strain root mean squared error (RMSE) may be due to temperature prediction errors. Future work improving temperature prediction could benefit the proposed model.

**Figure 6:**
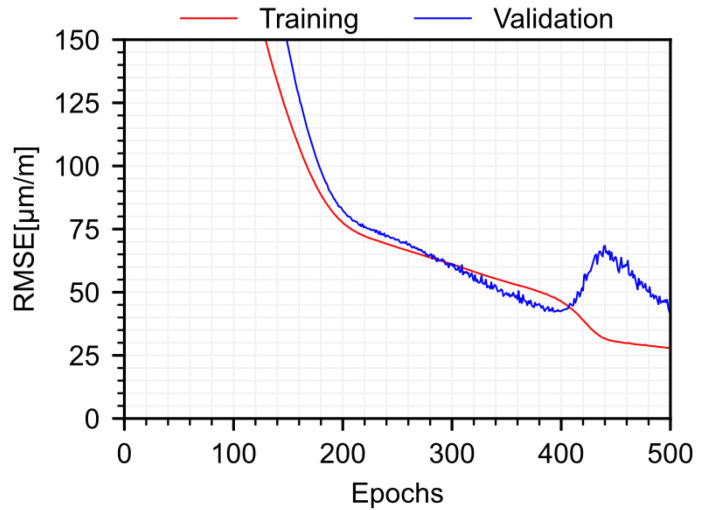
Training and validation losses for the total strain model.

The strain model is trained for 500 epochs with a learning rate of 0.001, using the Adam optimizer. The training and validation losses of the strain model are shown in Figure 6. The validation loss curve is unusual in that it presents a temporary loss of generalization between 400 and 450 epochs. The long-term strain associated with creep and shrinkage and seasonal thermal strain vary at similar rates during the first six months of the structure. Because only one year of data are used for training, the model has limited information to distinguish between these two strain components, so there is an interplay between the improvement of these two components as the training process progresses. The accelerated training loss observed between 400 and 420 epochs corresponds largely to adjustments to the long-term strain component to better fit the training data, but that temporarily reduces the generalization to the validation data. However, because the training data can be still be better fit by fine adjustment of the thermal and strain components, generalization starts to be recovered, as observed by the decrease in validation loss after 450 epochs.

**Figure 7:**
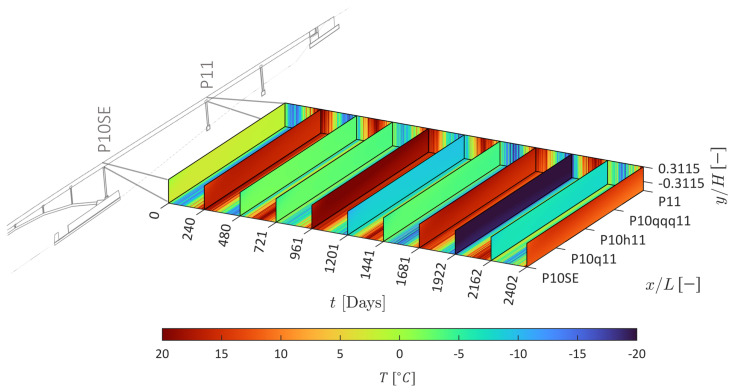
Predicted temperature field.

**Figure 8:**
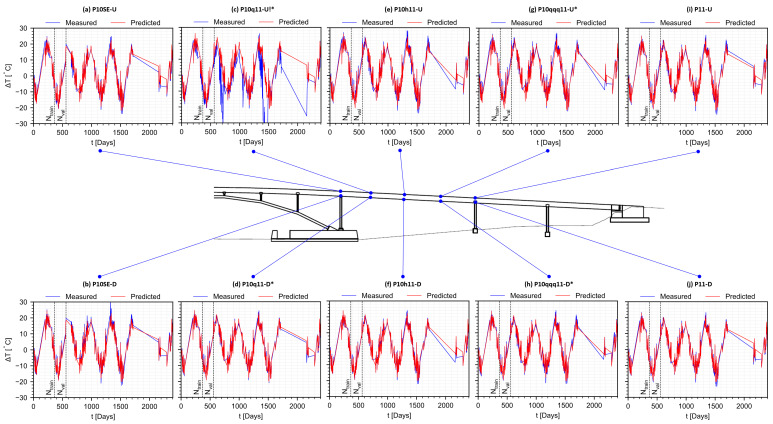
Predicted temperature at instrumented positions. Exclamation indicates positions with data anomaly. Asterisk indicates positions not observed during training or validation.

The predicted evolution of the temperature field $\Delta T^{x,t}$ over multiple years is shown in Figure 7, where the expected seasonal and daily variations are observed over time. The temperature predictions at observed and unobserved instrumented positions in the structure are shown in Figure 8. Good agreement is obtained at multiple positions in the structure, including those at unobserved positions (Figure 8d,g,h). However, a data anomaly is present at position P10q11-U (see Figure 8c), as unrealistic low temperatures are recorded, indicating a faulty temperature sensor. In such case, the methodology presented here can be used to substitute the data from the faulty sensor. Furthermore, although not shown in Figure 8, temperatures can be predicted for any day of interest such that the temperature model can be used for temperature data imputation. The apparent CTE is η=8.8 με${/}^{{}^{\circ}}$C, within typical values for concrete, and multiplying the temperature field $\Delta T^{x,t}$ by η gives the temperature-dependent strain (not shown here since it is only a scaled version of the temperature field).

**Figure 9:**
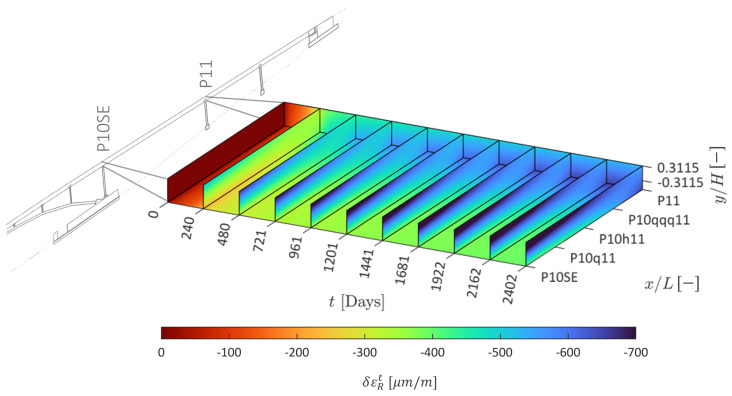
Predicted normal rheological strain field.

The predicted evolution of the normal rheological strain field $\delta \varepsilon_{R}^{x,t}$ is shown in Figure 9. Notice that there is an asymmetry in the evolution of the rheological strain that shows as a larger (in absolute terms) evolution closer to the upper region of position P10SE. The prediction reveals differential rheological effects both across the beam depth and over the span, which would not be captured by average cross-section models.

**Figure 10:**
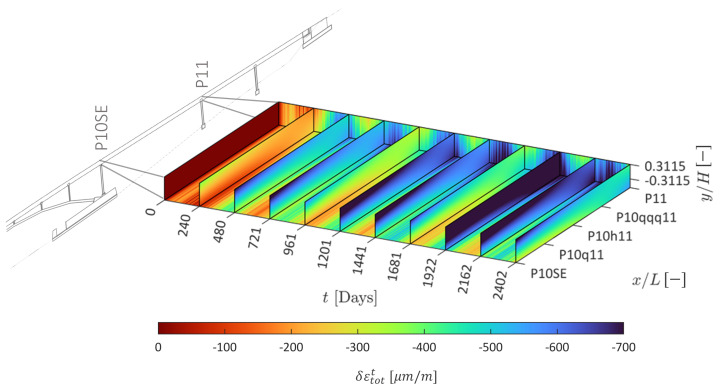
Predicted normal total strain field.

**Figure 11:**
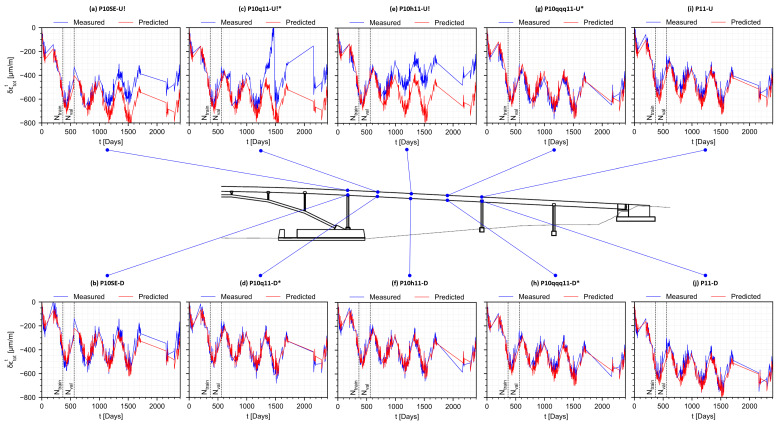
Predicted normal total strain at instrumented positions. Exclamation indicates positions with data anomaly. Asterisk indicates positions not observed during training or validation.

Adding the predicted rheological strain field and temperature-dependent strain field gives the predicted total strain field shown in Figure 10. The total strain predictions at observed and unobserved instrumented positions in the structure are shown in Figure 11. Good agreement is obtained at multiple positions in the structure (e.g., Figure 11f,i,j), including those at unobserved positions (e.g., Figure 11d,g,h). These results show that physics-informed data-driven prediction of differential creep and shrinkage effects mixed with aging and steel relaxation is feasible for prestressed concrete bridges, even at positions unobserved during training and validation. Because the strain measurement is compensated using the temperature measurement, anomalies in the temperature measurement are propagated into the strain data, as in the case of position P10q11-U (see Figure 11c), where large variations in strain are recorded due to the faulty temperature sensor. Using the predictions from the temperature model, it is in principle possible to correct the data from this strain sensor, although this is not performed here as it is out of the scope of this work.

Significant deviations from the predicted total strain are seen at P10SE-U (Figure 11a) and P10h11-U (Figure 11e), where relaxation is observed in the measured total strain. Because creep and shrinkage cause strain to become more negative over time, relaxation (i.e., trend towards positive strain) without significant load change is unexpected [2]. These findings are in agreement with long-term anomalies found in a previous work addressing the strain at the centroid of the same structure [35], where we show that prestress losses, a plausible source of long-term load change [10], are not sufficient to explain the relaxation observed, and that other mechanisms must be involved. In addition, in the context of all sensors, relaxation is observed only at P10SE-U (Figure 11a) and P10h11-U (Figure 11e), and possibly at P10q11-U (Figure 11c), which are all located on the top and left-half segment of the bridge. In a previous work, Ref. [33] shows that the connection to the main span at P10 exhibits lower stiffness than expected, and that the effects of this lower stiffness are markedly noticeable on the left-half segment of the bridge, which could explain why these anomalies are not manifest to the right of the midspan. Furthermore, a combination of negative bending with pulling could explain why these anomalies are seen at the top, but not at the bottom, as the stress distribution of these combined loads would be higher at the top and small at the bottom. For these reasons, the observed deviations correspond to either long-term degradation process or malfunction of strain sensors. Full diagnosis of the anomalies is currently under investigation but may include factors such as: Redistribution of prestress forces; Settlement of foundations, causing bending and pulling; Long-term impact of early age thermal cracking; and Concrete age mismatch at the P10 connection, since the main span was constructed prior to the southeast leg.

The RMSEs for training, validation, and test data are summarized in Table 2 for both the temperature and strain models. With the exception of the faulty temperature sensor, RMSEs in the test temperature data are typically under 3 ${}^{{}^{\circ}}$C, which is under 10% of the seasonal temperature variations. Except at positions exhibiting anomalous behavior, the RMSEs in the test strain data are typically under 60 µm/m, which is also under 10% of the magnitude of the rheological strain measured at multiple positions in the structure, and below the limits of concrete yield and ultimate compressive strains.

**Figure 12:**
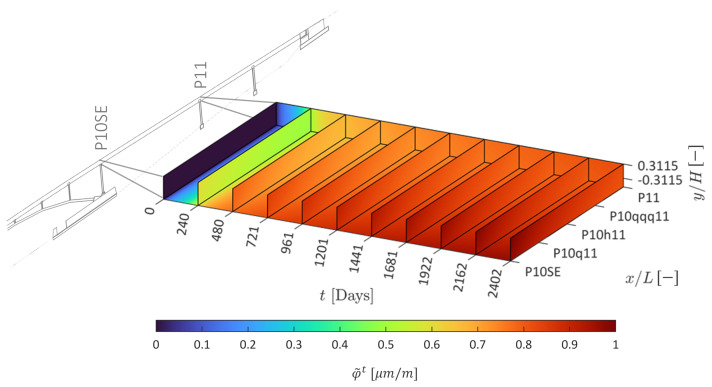
Evolution of the normalized rheological coefficient.

The evolution of the normalized rheological coefficient (see Equation (35)) is shown in Figure 12. It suggests that rheological effects closer to P10SE develop relatively more.

**Figure 13:**
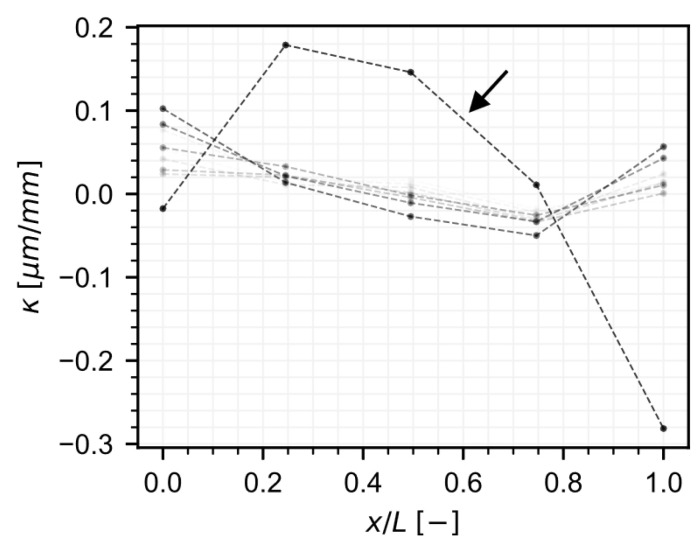
Curvature over time (lighter tones correspond to earlier times). Arrow highlights curvature after prestressing and form removal.

**Figure 14:**
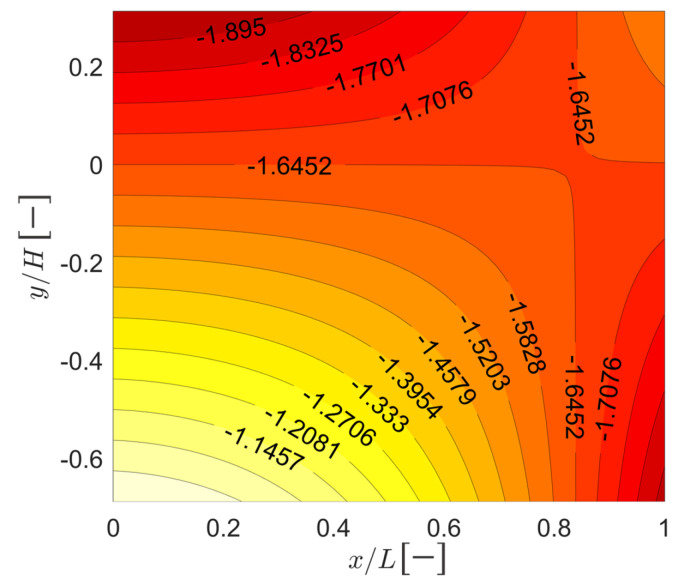
Normalized apparent static strain.

An interesting result appears looking at the apparent static strain (see Equation (34)). At a given position along the span, let $\varepsilon_{u}^{t}$, $\varepsilon_{d}^{t}$ denote the measured strain at time *t* in the up and down sensors, respectively. Then, the curvature at the given position can be calculated as

(46) $$\kappa^{t}=\frac{\varepsilon_{d}^{t}-\varepsilon_{u}^{t}}{h_{ud}},$$

where $h_{ud}$ is the distance between the up and down sensors. Figure 13 shows the evolution of the curvature from setting to until a few hours after form removal. As expected, a positive curvature is observed close to the midspan, and negative curvatures are observed at the supports. Furthermore, because the segment is prestressed, this indicates that, at the supports, the concrete is under less compression at the upper region of the cross-section right after form removal. Due to creep, one might expect the curvature to become more accentuated over time, as regions under more compression are expected to experience larger compressive creep strains. That is, the expectation is that the change in curvature

(47) $$\delta \kappa^{t}=\kappa^{t}-\kappa^{t^{\prime \prime }}=\frac{\delta \varepsilon_{d}^{t}-\delta \varepsilon_{u}^{t}}{h_{ud}},$$

would show, over time, negative values at the supports. However, the normalized apparent static strain, shown in Figure 14, reveals that close to P10SE (x/L=0) compression is higher at the upper region contrary to the expectation. The apparent static strain suggests that, over the first year (the period of the data used to fit the model), a positive change in curvature is observed at P10SE. Over the long-term, this is indeed the case at position P10SE. Notice that the total strain at P10SE-U is below the total strain at P10SE-D. We emphasize that average cross-section models, or predictions performed at the centroid only, would not reveal this. This unexpected long-term trend may be detected by careful analysis of the sensor data; for example, a previous work detected loss of stiffness close to P10SE [33]. However, the apparent static strain can be useful in revealing in a concise way unexpected trends present in the data of multiple sensors, highlighting the importance of models incorporating data from all sensors at once for training.

## 4. Conclusions

In this work, a physics-informed data-driven model for prediction of long-term normal strain field taking into account all instrumented positions at once in a concrete structure was presented. To the best of the author’s knowledge, this is the first work addressing prediction of strain fields over multiple years. In contrast to most of the literature, this new model inherently reveals differential rheological behavior across both the span and the beam depth. The method employs a simplified analytical model of the structure, together with a data-driven model to predict the temperature field in the structure, and incorporates position dependency on coefficients governing the evolution of the time-dependent behavior of the structure by the embedding of NNs.

Although the model is trained on the total strain data, after training, a decomposition of the total strain in a temperature-dependent component and a rheological component is obtained. Furthermore, the apparent static strain and rheological coefficients forming the rheological strain component can be further decoupled, revealing long-term trends, sometimes unexpected, as in the case of the curvature at P10SE as previously discussed, and this is in alignment with findings of previous studies on the same structure. Diagnosis of long-term anomalies identified will be the subject of future work.

It is shown that good prediction accuracy of both temperature and total strain can be obtained, enabling anomaly detection, as discussed in the Results section. The physics-based assumptions guiding the development of the model enabled the accurate prediction of the total strain even at unobserved positions. A future extension of the method is to integrate the notion of equivalent static strain and notional rheological coefficient with FEM of monitored structures to bypass the need of numerical time iteration in the prediction of long-term behavior of concrete structures.

## Figures and Tables

**Table 1:** Fiber optic sensor properties.

Property	Value
Strain uncertainty	2 µm/m
Temperature uncertainty	0.2 ${}^{{}^{\circ}}$C
Typical gauge length	60 cm
Dynamic range	−5000 to +7500 µm/m
Max. sampling frequency	250 Hz

**Table 2:** Root mean squared prediction errors. Exclamation indicates positions with data anomaly. Asterisk indicates positions not observed during training or validation.

	ΔT [${}^{{}^{\circ}}$C]			$\delta \varepsilon_{\mathrm{tot}}$ [µm/m]		
Position	Train	Val.	Test	Train	Val.	Test
P10SE-U ${}^{!}$	1.5	1.2	2.6	23.0	49.5	124.2
P10SE-D	1.1	1.3	2.7	33.0	38.9	51.4
P10q11-U ${}^{!}$*	2.5	2.1	13.8	27.2	48.3	174.9
P10q11D *	1.0	1.3	2.1	20.0	26.3	27.8
P10h11-U ${}^{!}$	1.3	2.1	2.7	34.9	22.1	174.5
P10h11D	0.9	1.0	2.2	23.0	27.6	27.1
P10qqq11-U *	1.5	2.1	2.5	37.0	18.7	36.9
P10qqq11D *	1.0	1.2	2.4	14.3	47.4	36.6
P11-U	1.0	1.5	2.5	29.2	67.8	49.0
P11D	1.0	1.1	2.3	21.7	59.5	41.6

## Data Availability

The data presented in this study are available on request from the corresponding author. The data are not publicly available due to privacy.

## Abbreviations

The following abbreviations are used in this manuscript:

MDPI	Multidisciplinary Digital Publishing Institute
FEM	Finite Element Method
SHM	Structural Health Monitoring
NN	Neural Networks
FOS	Fiber Optics Sensor
FBG	Fiber Bragg Grating
CNN	Convolutional Neural Networks
CTE	Coefficient of Thermal Expansion
FC	Fully Connected
RMSE	Root Mean Squared Error

## References

[1] Mehta P.K., Monteiro P.J.M. (2013). Concrete Microstructure, Properties, and Materials.

[2] Bazant Z.P., Jirasek M. (2018). Creep and Hygrothermal Effects in Concrete Structures.

[3] Dyer T. (2019). Concrete Durability.

[4] Melchers R.E. (2020). Long-Term Durability of Marine Reinforced Concrete Structures. J. Mar. Sci. Eng..

[5] Bonopera M., Chang K.C., Chen C.C., Lee Z.K., Sung Y.C., Tullini N. (2019). Fiber Bragg Grating-Differential Settlement Measurement System for Bridge Displacement Monitoring: Case Study. J. Bridge Eng..

[6] D’Altri A.M., Miranda S.D., Castellazzi G., Sarhosis V., Hudson J., Theodossopoulos D. (2020). Historic Barrel Vaults Undergoing Differential Settlements. Int. J. Archit. Herit..

[7] Ghorbani E., Svecova D., Thomson D.J., Cha Y.J. (2022). Bridge pier scour level quantification based on output-only Kalman filtering. Struct. Health Monit..

[8] Deng L., Cai C.S. (2010). Bridge Scour: Prediction, Modeling, Monitoring, and Countermeasures—Review. Pract. Period. Struct. Des. Constr..

[9] Glisic B., Inaudi D., Lau J., Fong C. (2013). Ten-year monitoring of high-rise building columns using long-gauge fiber optic sensors. Smart Mater. Struct..

[10] Abdel-Jaber H., Glisic B. (2019). Monitoring of long-term prestress losses in prestressed concrete structures using fiber optic sensors. Struct. Health Monit..

[11] ACI Committee 209 (2008). Guide for Modeling and Calculating Shrinkage and Creep in Hardened Concrete. www.civil.northwestern.edu/people/bazant/PDFs/Backup%20of%20Papers/R21.pdf.

[12] CEB-FIP (1990). CEB-FIP Model Code 1990: Design Code.

[13] Gardner N., Lockman M. (2001). Design provisions for drying shrinkage and creep of normal-strength concrete. ACI Mater. J..

[14] Hubler M., Wendner R., Bazant Z. (2015). Statistical justification of model B4 for drying and autogenous shrinkage of concrete and comparisons to other models. Mater. Struct..

[15] Al-Kamyani Z., Guadagnini M., Kypros P. (2018). Predicting shrinkage induced curvature in plain and reinforced concrete. Eng. Struct..

[16] Kaklauskas G., Gribniak V. (2011). Eliminating Shrinkage Effect from Moment Curvature and Tension Stiffening Relationships of Reinforced Concrete Members. J. Struct. Eng..

[17] Sousa H., Santos L., Chryssanthopoulous M. (2019). Quantifying monitoring requirements for predicting creep deformations through Bayesian updating methods. Struct. Saf..

[18] Joint ACI-ASCE Committee 423 (2016). Guide to Estimating Prestress Losses. https://www.doc88.com/p-18661732142167.html.

[19] Sousa C., Sousa H., Neves A.S., Figueiras J. (2012). Numerical Evaluation of the Long-Term Behavior of Precast Continuous Bridge Decks. J. Bridge Eng..

[20] Sousa H., Bento J., Figueiras J. (2013). Construction assessment and long-term prediction of prestressed concrete bridges based on monitoring data. Eng. Struct..

[21] Abdellatef M., Vorel J., Wan-Wendner R., Alnaggar M. (2019). Predicting Time-Dependent Behavior of Post-Tensioned Concrete Beams: Discrete Multiscale Multiphysics Formulation. J. Struct. Eng..

[22] Alnaggar M., Cusatis G., Di Luzio G. (2013). Lattice Discrete Particle Modeling (LDPM) of Alkali Silica Reaction (ASR) deterioration of concrete structures. Cem. Concr. Compos..

[23] Alnaggar M., Di Luzio G., Cusatis G. (2017). Modeling Time-Dependent Behavior of Concrete Affected by Alkali Silica Reaction in Variable Environmental Conditions. Materials.

[24] Ghamsemzadeh F., Manafpour A., Sajedi S., Shekarchi M., Hatami M. (2016). Predicting long-term compressive creep of concrete using inverse analysis method. Constr. Build. Mater..

[25] Han B., Xiang T.Y., Xie H.B. (2017). A Bayesian inference framework for predicting the long-term deflection of concrete structures causeed by creep and shrinkage. Eng. Struct..

[26] Bal L., Buyle-Bodin F. (2013). Artificial neural network for predicting drying shrinkage of concrete. Constr. Build. Mater..

[27] Hauge M. (2019). Machine Learning for Predictions of Strains Due to Long-Term Effects and Temperature in Concrete Structures. Master’s Thesis.

[28] Che Z., Purushotham S., Cho K. (2018). Recurrent Neural Networks for Multivariate Time Series with Missing Values. Sci. Rep..

[29] Che Z., Purushotham S., Li G., Jiang B., Liu Y. Hierarchical deep generative models for multi-rate multivariate time series. Proceedings of the International Conference on Machine Learning.

[30] Rubanova Y., Chen R.T.Q., Duvenaud D.K. (2019). Latent Ordinary Differential Equations for Irregularly-Sampled Time Series. Adv. Neural Inf. Process. Syst..

[31] Oh B., Park H., Glisic B. (2021). Prediction of long-term strain in concrete structure using convolutional neural networks, air temperature and time stamp of measurements. Autom. Constr..

[32] Karniadakis G., Levrelodos I., Lu L., Perdiaris P., Wang S., Yang L. (2021). Physics-informed machine learning. Nat. Rev. Phys..

[33] Sigurdardottir D., Glisic B. (2015). On-Site Validation of Fiber-Optic Methods for Structural Health Monitoring: Streicker Bridge. J. Civ. Struct. Health Monit..

[34] Glisic B. (2019). CEE 537 Structural Health Monitoring, Graduate Course. https://cee.princeton.edu/people/branko-glisic.

[35] Pereira M., Glisic B. (2022). A hybrid approach for prediction of long-term behavior of concrete structures. J. Civ. Struct. Health Monit..

[36] Montavon G., Orr G., Muller K.R. (2012). Neural Networks: Tricks of the Trade.

[37] Kingma D., Ba J. Adam: A method for stochastic optimization. Proceedings of the 3rd International Conference for Learning Representations.

